# Tricuspid Regurgitant Jet Velocity Point-of-Care Ultrasound Curriculum Development and Validation

**DOI:** 10.24908/pocus.v6i2.15190

**Published:** 2021-11-23

**Authors:** Zachary W Binder, Sharon E O"Brien, Tehnaz P. Boyle, Howard J Cabral, Joseph R Pare

**Affiliations:** 1 UMass Memorial Medical Center, University of Massachusetts Medical School Worchester, MA USA; 2 Boston Medical Center, Boston University School of Medicine Boston, MA USA; 3 Boston University School of Public Health MA, Boston USA

**Keywords:** tricuspid regurgitant jet velocity, point-of-care ultrasound, novice echocardiography, pulmonary hypertension

## Abstract

**Introduction: **The American College of Emergency Physicians (ACEP) recommends that Emergency Medicine physicians with advanced training can evaluate right ventricular (RV) pressures via point-of-care ultrasound (POCUS) by measuring a tricuspid regurgitant jet (TRJ). We were unable to find a published curriculum to deliver education for this at any skill level. Therefore, we developed, delivered, and evaluated a curriculum for the assessment of TRJ for novice physician sonographers. **Methods: **We designed an educational intervention for novice physician sonographers. The curriculum was created using a modified Delphi methodology. All novice sonographers participated in the educational intervention which consisted of a didactic lecture followed by hands-on-deliberate practice on healthy medical student volunteers with expert feedback in a simulated setting. Sonographer’s knowledge was assessed at 3 time points: pre-intervention, immediately post-intervention, and 3 months post-intervention (retention assessment) by multiple choice exam. **Results: **Nine novice physician sonographers participated in the intervention. Mean exam performance increased from 55.6% [standard deviation (SD) 11.3%] on the pre-intervention exam to 94.4% (SD 7.3%) on the post-intervention exam and 92.9% (SD 12.5%) on the retention exam. The mean improvement between the pre- and post- exam was +38.9% (95% CI 31.8 - 46.0), and between the pre-exam and retention exam +37.1% (95% CI 22.3 - 52.0). **Conclusion: **Sonographer knowledge of TRJ assessment improved following a brief educational intervention as measured by exam performance. Given the expanding role of POCUS it is increasingly important to provide effective resources for teaching these skills. This work establishes the basis for further study and implementation of our TRJ curriculum.

## Background

Point-of-care ultrasound (POCUS) has emerged as an integral aspect of emergency care and therefore recognized by the Accreditation Council for Graduate Medical Education (ACGME) and American College of Emergency Physicians (ACEP) as a core educational requirement 1, 2. ACEP’s policy statement “The Core Content of Clinical Ultrasonography Fellowship Training” states “for situations where there is a concern for increased right ventricular (RV) pressure (i.e. acute pulmonary embolism), Continuous Wave Doppler may be used to estimate RV systolic pressure…by measuring the peak velocity of the tricuspid regurgitant jet (TRJ)” 3. Tricuspid regurgitant jet velocity can serve as an estimate for RV systolic pressure by using the simplified Bernoulli equation: RV systolic pressure = 4V^2 ^+ estimated right atrial pressure, where V is the peak TRJ velocity 4. POCUS serves as a safe and useful diagnostic test to estimate RV systolic pressure which can obviate the need for invasive cardiac procedures.

Although completing a fellowship is one way to gain this knowledge, it is likely that general Emergency Medicine physicians might also have interest in developing this competency. In addition to these policy statements, there has been interest in emergency physicians’ ability to assess for pulmonary hypertension in the Emergency Department (ED) setting. A published review on the evaluation and management of pulmonary hypertension in the ED recommends imaging should include a “chest radiograph and bedside echocardiography” 5. A case series emphasizes the importance of cardiac POCUS with 8 pediatric patients who received an initial diagnosis of pulmonary hypertension by ED evaluation 6. 

Despite the ACEP policy that Emergency Medicine physician’s with advanced ultrasound training should consider evaluation of RV pressures by POCUS, and research suggesting the importance of emergency physician’s (EP) ability to perform this assessment, we were unable to find a published curriculum to deliver education for this skill at any learner level. Studies have shown that EP sonographers can learn to accurately perform various aspects of a point-of-care echocardiogram through didactic instruction and practical training 7, 8, 9, 10, 11, 12. 

### Objectives

We created a curriculum for evaluation of RV pressure by POCUS assessment of tricuspid regurgitant jet velocity (TRJV) that was delivered to a group of novice physician sonographers. The educational intervention was assessed by comparing sonographer scores on a pre-intervention exam, post-intervention exam, and a retention exam at 3 months. 

## Methods

We assessed an educational intervention as part of a larger prospective cross-sectional study. The prospective study tested the feasibility of novice physician sonographers to perform echocardiograms of adequate quality to exclude TRJV pathology in Emergency Department patients 13. The educational intervention was performed in an urban tertiary care level 1 trauma center with an accredited Emergency Medicine residency and Pediatric Emergency Medicine (PEM) fellowship. The Boston University Medical Campus and Boston Medical Center Institutional Review Board approved this work. Study participants provided informed verbal consent.

### Novice Physician Sonographer Population

We designed the educational intervention to be delivered to novice physician sonographers. Based on the ACEP policy statement we included novice physician sonographers as having performed fewer than 50 echocardiograms 2. All participants had completed an introductory ultrasound orientation which included basic cardiac ultrasound. Emergency Medicine Interns, Pediatric Emergency Medicine Fellows, and Pediatric Emergency Medicine Attendings were recruited by the principle investigator (PI) as unpaid volunteers. Prospective participants were first contacted by email, followed by in-person enrollment if interested. 

### Curriculum Development

The intervention’s PI and primary cardiologist created a curriculum as described. A focused literature review led to the generation of a preliminary curriculum 14, 15, 16. The proposed curriculum contained four elements: (1) obtaining an apical 4-chamber view, (2) positioning the color box, (3) optimizing the TRJV color signal, and (4) interrogating with continuous-wave Doppler (Video S1). Each element was subdivided into several critical action steps. The curriculum was then revised and validated using modified Delphi methodology 17, 18. The curriculum was distributed to 3 cardiologists who were otherwise unaffiliated with the intervention. The panel of cardiologists participated in multiple rounds of review and revision. We determined that consensus had been reached when 100% of experts agreed on the main curricular elements and when a majority agreed on each critical action step. The final curriculum contained the 4 main elements and 14 critical action steps (Table 1). The curriculum served as the central document from which all other intervention documents were generated (exams, didactic lecture, grading rubric). 

**Table 1:** Tricuspid Regurgitant Jet Velocity Curriculum

**Curricular Element**	**Critical Action Step**
Apical 4-Chamber View	Image orientation with cardiac apex at the top of screen and left ventricle to the right of screen
	Outline of all four chambers simultaneously visualized
	Image aligned with ultrasound beam parallel to intraventricular septum and perpendicular to Tricuspid Valve
	Image saved of apical 4-chamber view
Color Box Positioning	Color box extending from the back wall of right atrium past the tricuspid valve leaflet tips
	Color box width minimized to just include tricuspid valve orifice
TRJV Color Signal Optimization	Clip saved showing a dynamic sweep through the Tricuspid Valve (anterior → posterior) or (posterior → anterior)
	Select probe position that generates maximal regurgitant color signal
Continuous Wave Doppler	Doppler cursor placed in the middle of tricuspid regurgitant color jet
	Doppler cursor aligned parallel to color jet flow
	Doppler gain adjusted to maximize waveform
	Baseline adjusted to maximize display of wave form
	Image includes three full cardiac cycles
	Image saved with continuous-wave Doppler applied
TRJV = Tricuspid Regurgitant Jet Velocity. Reproduced with permission from: Binder ZW, O’Brien SE, Boyle TP, et al. “Novice Physician Ultrasound Evaluation of Pediatric Tricuspid Regurgitant Jet Velocity”. Western Journal of Emergency Medicine. 2020; 21(4): 1029-1035	

### Assessment

Exam questions were developed to provide an objective assessment of the educational intervention. Questions were multiple-choice and correlated to the TRJ curriculum. The questions were developed by the intervention’s PI before undergoing multiple rounds of revision and final approval by a group of content experts in the fields of cardiology and POCUS. This group of experts was distinct from the panel of cardiologists who validated the TRJ curriculum. A total of 20 exam questions were created. Exams were administered at three time points: prior to the educational intervention (pre-intervention assessment), immediately after the educational course (post-intervention assessment), and 3 months after the educational course (retention assessment) (Table 2). Each of the 3 exams contained 10 questions. (Document S1, S2, S3). The pre-intervention exam and post-intervention exam were unique, whereas the retention exam was a random selection of 10 questions from the question bank.

**Table 2:** Educational Intervention Format

**Educational Component**	**Duration**	**Content**
Pre-Exam	15 minutes	10 multiple choice questions
Didactic Lecture	30 minutes	Still image and video clip based
Hands-On Workshop	150 minutes	Deliberate practice with expert feedback
Post-Exam	15 minutes	10 multiple choice questions
Scanning Window	3 months	Prospective intervention study 13
Retention Exam	15 minutes	10 multiple choice questions

### Curriculum Delivery

The educational intervention consisted of a 3-hour course that included a didactic lecture (30 minutes) and hands-on workshop (150 minutes) (Table 2). The didactic lecture was derived from the TRJV curriculum and taught sonographers the steps required to obtain TRJV images through still image and video clip modalities. The lecture was prepared and delivered by the intervention’s PI and primary cardiologist. The didactic lecture was modified for the purposes of publication and is included in the supplemental materials (Document S4), along with a video clip of a complete TRJ ultrasound being performed (Video S1).

A hands-on workshop immediately following the didactic lecture consisted of deliberate practice with direct expert feedback. The novice physician sonographers were divided into two groups of four or five participants led by an expert instructor - the primary intervention cardiologist or a professional certified cardiac sonographer. Novice physician sonographers first observed the expert perform image acquisition. This was followed by hands-on practice and the opportunity to observe other novices as they attempted to acquire images with expert guidance. Each sonographer practiced image acquisition on four human models and received guided feedback from each of the instructors. Each sonographer completed a minimum of 5 practice scans during the workshop followed by one graded scan. The instructors used a developed image grading rubric to grade scans (Document S5) 13. Scans received a maximum score of 4 with each element receiving a complete (1), incomplete (0) or not performed (0). A passing grade required a score of 4 out of 4. Novice physician sonographers who were unable to pass on their initial attempt received additional focused feedback and time to practice until they were able to perform a passing scan. The graded scans established the novice’s ability to obtain TRJ images. 

### Ultrasound Models

All ultrasound models used during the educational intervention were healthy medical student volunteers. All models were initially recruited through an advertisement in the medical school e-newsletter. Models were paid a financial stipend. There was a certified cardiologist on hand at all times during the educational intervention who was prepared to privately disclose any incidental findings to the volunteer models who would then be encouraged to follow-up with their primary care physician. No incidental findings were discovered during the hands-on session.

### Statistical Analysis 

Descriptive statistics and paired t-test were performed to analyze the data. All statistical analyses were performed using STATA v 13.1 (College Station, TX).

## Results

Nine novice physician sonographers participated in the educational intervention (3 Emergency Medicine Interns, 2 Pediatric Emergency Medicine Fellows, and 4 Pediatric Emergency Medicine Attendings). Five (55.5%) of the participants were female. Two of the sonographers (one Emergency Medicine Intern, and one Pediatric Emergency Medicine Attending) dropped out after the educational intervention due to time constraints. They did not complete the retention exam.

At the conclusion of the hands-on session, all participating sonographers successfully passed the test scan with a score of 4 out of 4 on their first attempt, within the allotted 3 hours. Table 3 shows sonographer exam results.

**Table 3:** Sonographer Exam Results.

**2.A Exam Scores (n=9)**			
	**Mean**	**SD**	**Range**
Pre-Exam	55.6%	+/- 11.3%	40-80%
Post-Exam	94.4%	+/- 7.3%	80-100%
Retention Exam *	92.9%	+/- 12.5%	70-100%
**2.B Score Change**			
	**% Change [95% CI]**	**SD**	**P value**
Pre-Exam→Post-Exam	+38.9 [+31.8 - +46.0]	+/- 9.3	0.0001
Pre-Exam→Retention Exam *	+37.1 [+22.3 - +52.0]	+/- 16.0	0.0001
SD = Standard Deviation; CI = Confidence Interval * n = 7			

## Discussion

A primary goal of this publication is to make available our educational intervention with exams to further the resources available for point of care ultrasound learners. The accompanied knowledge evaluation shows retention of this curriculum was strong even at 3 months. 

The intervention found a statistically significant improvement in sonographer exam performance following the educational intervention. This improvement was maintained after an interval period of 3 months. These results are consistent with prior studies that have shown emergency medicine physicians can accurately assess and measure components of a POCUS echocardiogram after educational interventions that include didactic instruction and hands-on training 7, 8, 9, 10, 11, 12. To our knowledge this intervention represents the first time that an educational intervention aimed at teaching TRJV has been tested. We believe this manuscript provides preliminary evidence that this educational intervention improves key knowledge regarding the principles and skills required for the evaluation of TRJV.

Obtaining expertise for a given POCUS application has multiple stages. One must first obtain the applicable knowledge, then learn to physically perform the scan, and finally learn to interpret their images in order to integrate clinically. Sonographers performed well on the post intervention assessment which tested accrued knowledge (initial stage). 

A previously published prospective research study by Binder et al. tested sonographer’s ability to physically perform TRJV scans (second stage) 13. During that study novice physician sonographers were graded by the study’s primary cardiologist using the same grading rubric as this educational intervention (**Document S5). **In that study novice sonographers obtained a satisfactory apical 4-chamber view in 85% (95% CI 77.1-92.9), positioned the color box accurately 65% (95% CI 54.5-75.5), optimized TRJV color signal 78.7% (95% CI 69.8-87.7), and optimized continuous-wave Doppler in 55% (95% CI 44.1-66.0) of echocardiograms 13. Future research into the implementation of this curriculum could compare novice performance to the gold standard of sonographer performed comprehensive echocardiogram to provide further validation of skill acquisition.

## Limitations

We focused on one method for assessing for pulmonary hypertension, tricuspid regurgitant jet velocity, in an apical 4-chamber window. The American Society of Echocardiography Guidelines suggests interrogating the tricuspid regurgitant jet velocity in multiple windows which was not included as part of this educational intervention.^4^ This intervention was not intended to teach all the components of a comprehensive cardiac point-of-care ultrasound. It should also be noted that the use of tricuspid regurgitant jet velocity as an estimate for RV systolic pressure requires estimation and addition of the right atrial pressure. Complete evaluation should also assess for obstruction at the pulmonic valve and RV outflow tract which was not done during this novice educational intervention.^4^

Additional echocardiographic findings such as “right atrial enlargement, RV dilatation, increased RV free wall thickness, end-systolic flattening of the intraventricular septum, and interventricular interdependence visualized as a ‘D’- shaped left ventricle in diastole” can be used to aid the diagnosis of pulmonary hypertension 5. These additional techniques were not taught nor tested as part of this curriculum. Additionally, the curriculum did not incorporate pulsed-wave Doppler prior to continuous-wave Doppler analysis. The risk in excluding pulsed-wave Doppler is overestimating the TRJV through contamination of signal from extremely rare intra-cardiac shunting lesions. This is an accepted practice in echocardiography and has been used in prior studies on the topic 19. 

This evaluation of the educational intervention was limited to 7 participants. We hope to replicate our results on a larger sample of participants in the future.

## Conclusions

Based on national recommendations that emergency medicine physicians with advanced ultrasound training are expected to learn, perform and interpret a TRJV as part of POCUS echocardiography we chose to develop a curriculum to teach this skill to novices. The curriculum was able to greatly improve knowledge as tested by multiple choice exam, however capability of hands-on skills varied. This work establishes the basis for further study and implementation of the TRJV curriculum. 

## Statement of Ethics Approval/Consent 

This study was approved and monitored by the institution’s IRB. All participants provided informed consent prior to participation.

## Disclosures

The study authors have no financial or other conflicts of interest in relation to this study.

## Supplementary Materials

Video S1 Video of complete tricuspid regurgitant jet study being performed.

Supplementary Document S1

Supplementary Document S2

Supplementary Document S3

Supplementary Document S4

Supplementary Document S5
