# Supplementary material for: Tricuspid Regurgitant Jet Velocity Point-of-Care Ultrasound Curriculum Development and Validation
Source: POCUS J. 2021 Nov 23;6(2):88–92. doi: 10.24908/pocus.v6i2.15190 (PMC9316333; doi:10.24908/pocus.v6i2.15190)
Supplement: Supplementary Document S1 [file pocusj-06-15190-s002.pdf]

Supplementary Documents

Pre-Intervention Exam

1. Which of the following is the correct ultrasound beam orientation for an apical 4-chamber view of the heart?
    A. Perpendicular to intraventricular septum and perpendicular to atrioventricular valves
    B. Parallel to intraventricular septum and perpendicular to atrioventricular valves
    C. Parallel to intraventricular septum and parallel to atrioventricular valves
    D. Perpendicular to intraventricular septum and parallel to atrioventricular valves

2. What is the lowest velocity <u>above</u> which a tricuspid regurgitant jet is pathologic?
    A. 1.5 m/s
    B. 1.8 m/s
    C. 2.5 m/s
    D. 3.3 m/s

3. Which of the following is <u>not</u> a required step in the process of measuring a tricuspid regurgitant jet?
    A. Applying color Doppler over the tricuspid valve
    B. Applying continuous wave Doppler over the tricuspid valve
    C. Placing measurement cursor at the peak of Doppler waveform
    D. Applying M-mode over the tricuspid valve

4. What is the error in the following attempt to visualize tricuspid regurgitation?

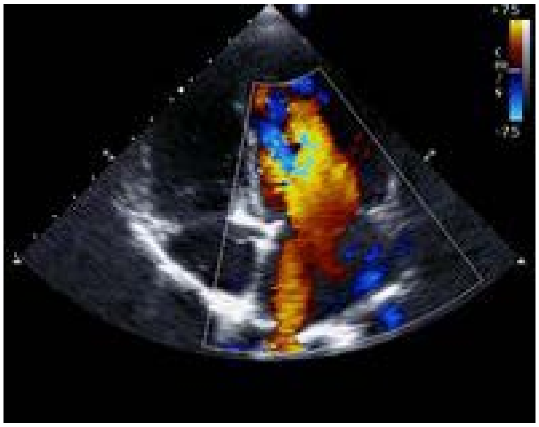

A. The ultrasonographer has obtained a parasternal short axis view of the heart instead of an apical 4-chamber view.
B. The ultrasonographer has focused the color box over the mitral valve instead of the tricuspid valve.
C. The ultrasonographer has focused the color box over the pulmonic valve instead of aortic valve.
D. The ultrasonographer has obtained a parasternal long axis view of the heart instead of subxiphoid view.

5. Which of the following is acceptable patient positioning for analysis of a tricuspid regurgitant jet?

A. Place patient in a slight Trendelenburg position.
B. Place patient in the right lateral decubitus position.
C. Place patient in the left lateral decubitus position.
D. Place patient in a slight reverse Trandelenburg position.

6. Which of the following is a component of a correctly sized color box when interrogating a tricuspid regurgitant jet?

A. Color box should include right ventricular apex
B. Color box should include aortic outflow tract
C. Color box should include base of right atrium
D. Color box should include intraventricular septum

7. Which of the following is a <u>not</u> a required step to correctly measure a tricuspid regurgitant jet?

A. The continuous wave Doppler cursor is placed perpendicular to color jet flow.
B. The Doppler gain is adjusted to optimize waveform visualization.
C. The measurement caliper is placed to exclude Doppler feathering.
D. The measurement caliper is placed at the apex of largest complete envelope.

8. Which of the following locations represents the correct location for Doppler cursor placement when measuring a Tricuspid Regurgitant jet?

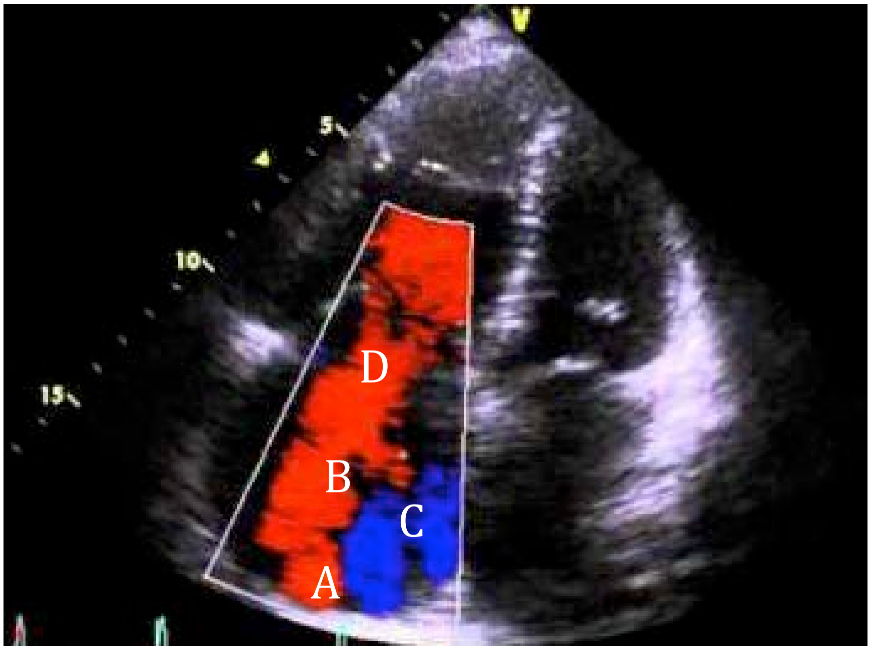

A. A
B. B
C. C
D. D

9. What is incorrect with the following attempt to measure a tricuspid regurgitant jet?

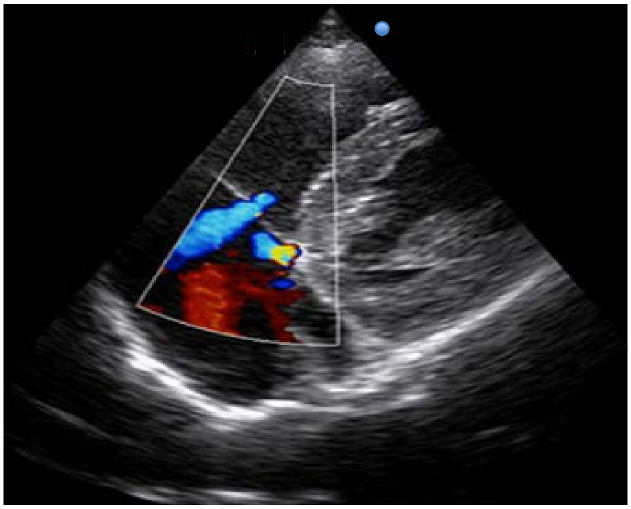

A. Color wave is interrogating incorrect valve
B. Ultrasound beam is aligned incorrectly
C. Color box width is inappropriate
D. Color box does not extend far enough into ventricle

10. Which of the following is the correct location for the measurement cursor when measuring tricuspid regurgitant jet on continuous wave Doppler?

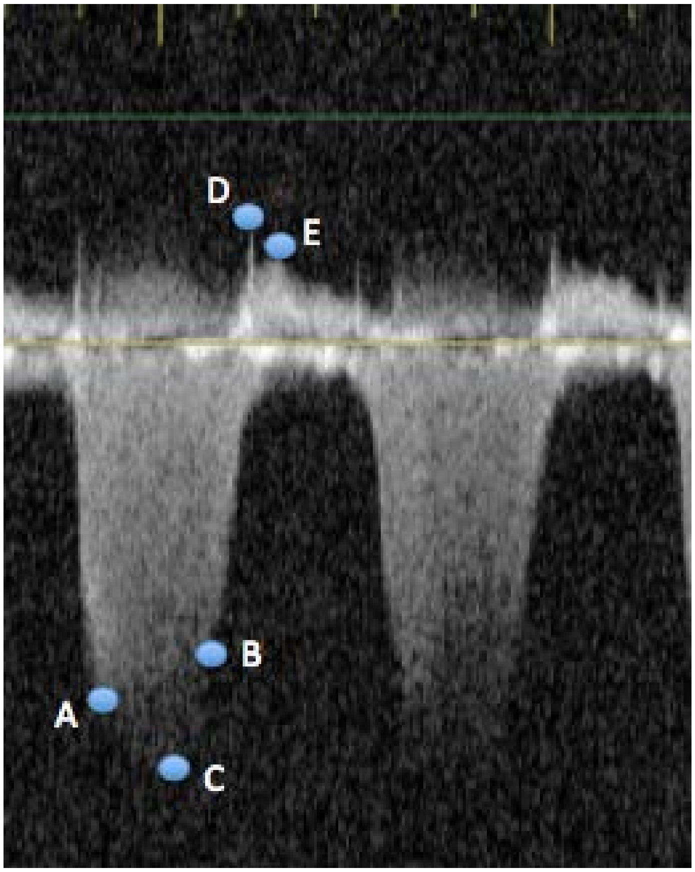

A. A
B. B
C. C
D. D
