# Supplementary material for: Tricuspid Regurgitant Jet Velocity Point-of-Care Ultrasound Curriculum Development and Validation
Source: POCUS J. 2021 Nov 23;6(2):88–92. doi: 10.24908/pocus.v6i2.15190 (PMC9316333; doi:10.24908/pocus.v6i2.15190)
Supplement: Supplementary Document S2 [file pocusj-06-15190-s003.pdf]

Post-Intervention Exam

1. Which of the following best represents the range of physiologic Tricuspid Regurgitant Jet velocities?
    1. 0 m/s - 2.5 m/s
    2. 0 m/s - 1.3 m/s
    3. 0 m/s - 3.3 m/s
    4. 0 m/s - 2.8 m/s

2. Which of the following is the correct location for the measurement cursor when measuring tricuspid regurgitant jet on continuous wave Doppler?

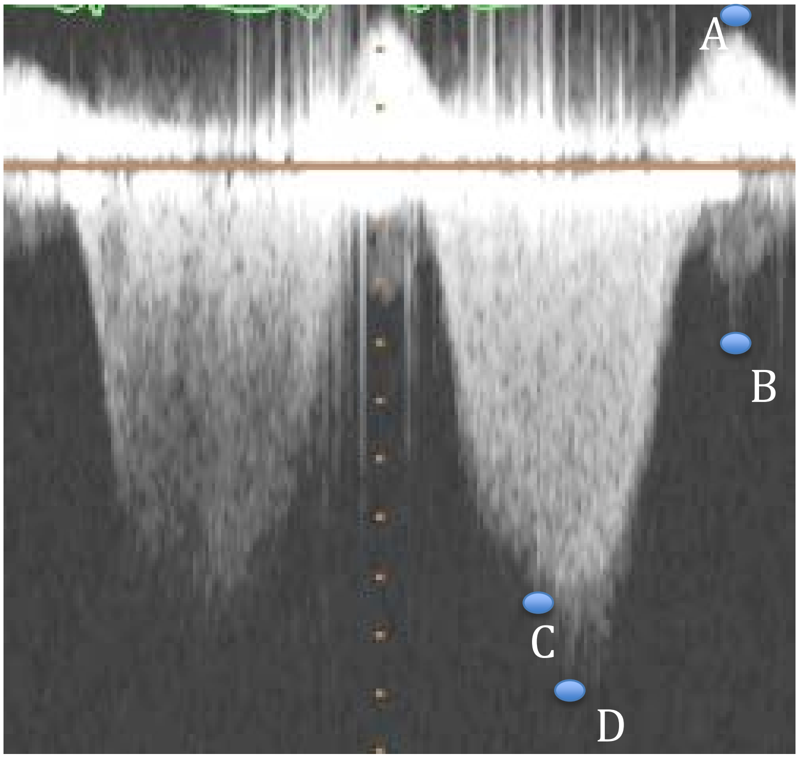

1. A
2. B
3. C
4. D

3. Which of the following ultrasound functions most reliably allows for the measurement of a tricuspid regurgitant jet at pathologic velocities?
    1. Power Doppler
    2. Color wave Doppler
    3. Continuous wave Doppler
    4. M-Mode

4. Which of the following locations represents the correct location for Doppler cursor placement when measuring a Tricuspid Regurgitant Jet?

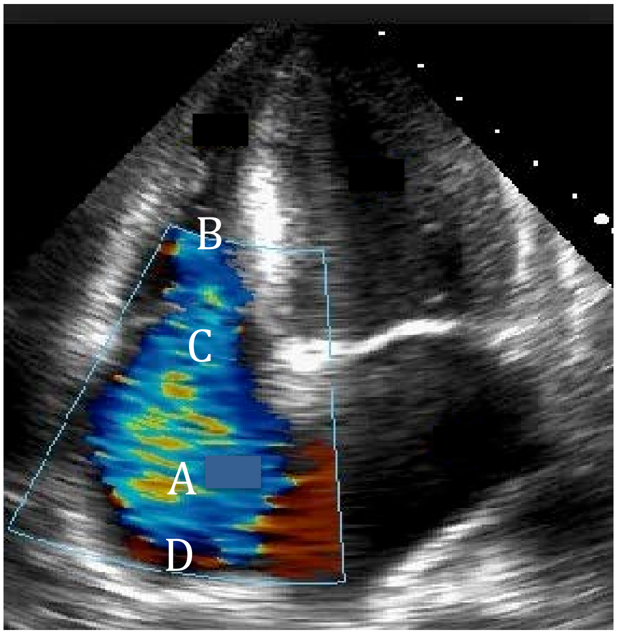

1. A
2. B
3. C
4. D

5. Which of the following statements regarding cardiac ultrasound is <u>false</u>?
   1. Color wave Doppler only measures movement in the direction of the ultrasound probe
   2. Continuous wave Doppler enables measurement of high velocity blood flow
   3. A smaller color box will generate a clearer continuous wave Doppler
   4. Continuous wave Doppler is a mode in which the transducer emits and receives the ultrasound beam continuously

6. Which of the following statements is correct regarding acquisition of an apical 4-chamber view of the heart?
   1. The intraventricular septum should appear horizontally on the screen
   2. The atrioventricular valves should be aligned vertically on the screen
   3. The ultrasound beam should be parallel to the atrioventricular valves
   4. The ultrasound beam should be parallel to the intraventricular septum

7. Which of the following maneuvers can <u>help facilitate</u> the accurate acquisition of a tricuspid regurgitant jet?

1. Placement of the patient's head of bed to + 30 degrees.
2. Place the patient in left lateral decubitus position.
3. Elevate the patient's legs on towel roll.
4. Ask the patient to hold their breath.

8. All of the following are true of a properly sized color box (when investigating for tricuspid regurgitation) <u>except:</u>

1. The color box must include the tricuspid valve leaflets.
2. The color box width should be minimized to improve Doppler quality.
3. The color box must include the intraventricular septum and extend into the left ventricle.
4. The color box should extend to the base of the right atrium.

9. What is the error in the following attempt to visualize tricuspid regurgitation?

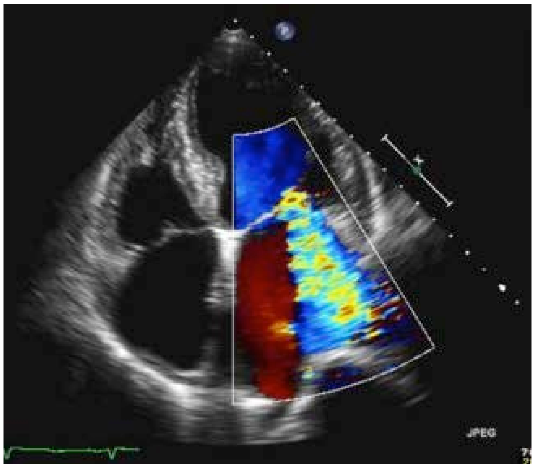

1. The ultrasonographer has obtained a parasternal short axis view of the heart instead of an apical 4-chamber view.
2. The ultrasonographer has focused the color box over the mitral valve instead of the tricuspid valve.
3. The ultrasonographer has obtained a subxiphoid view of the heart instead of a parasternal long axis view.
4. The ultrasonographer has focused the color box over the pulmonic valve instead of the mitral valve.

10. All of the following statements regarding Tricuspid Regurgitant Jet measurement are correct <u>except</u>:

1. Waveform visualization can be improved by adjusting the Doppler gain.
2. Doppler feathering should be included in a Tricuspid Regurgitant Jet measurement.
3. The measurement calipers should be placed at the apex of the largest complete envelope.
4. The continuous wave Doppler cursor should be oriented parallel to color flow
