# Supplementary material for: Tricuspid Regurgitant Jet Velocity Point-of-Care Ultrasound Curriculum Development and Validation
Source: POCUS J. 2021 Nov 23;6(2):88–92. doi: 10.24908/pocus.v6i2.15190 (PMC9316333; doi:10.24908/pocus.v6i2.15190)
Supplement: Supplementary Document S4 [file pocusj-06-15190-s005.pdf]

Novice Sonographer Assessment of Tricuspid Regurgitant Jet Velocity

Presented By:

Zachary Binder MD, Pediatric Emergency Medicine Fellow, Boston Medical Center

Sharon Obrien MD, Pediatric Cardiology, Boston Medical Center

Presentation Objectives

- Define Tricuspid Regurgitant Jet Velocity

- Learn the steps to obtaining and measuring a Tricuspid Regurgitant Jet Velocity

Clinical Implications

- Pulmonary HTN has **high morbidity and mortality**

- The ability to assess for pulmonary hypertension could assist in the management of the following presentations: **syncope, pulmonary embolism, asthma, sickle cell crisis**

Clinical Implications

- Tricuspid Regurgitant jet velocity (**TRJV**) is used by cardiologists to estimate **right ventricular systolic pressure via simplified Bernoulli equation**

RV systolic pressure = 4(TRJV)^2 + RA pressure

Tricuspid Regurgitant Jet Velocity

- Measured back flow of blood from the right ventricle into the right atria during systole

- **TRJV < 2.5 m/s is "present"**
- **TRJV > 2.5 m/s is "elevated"**

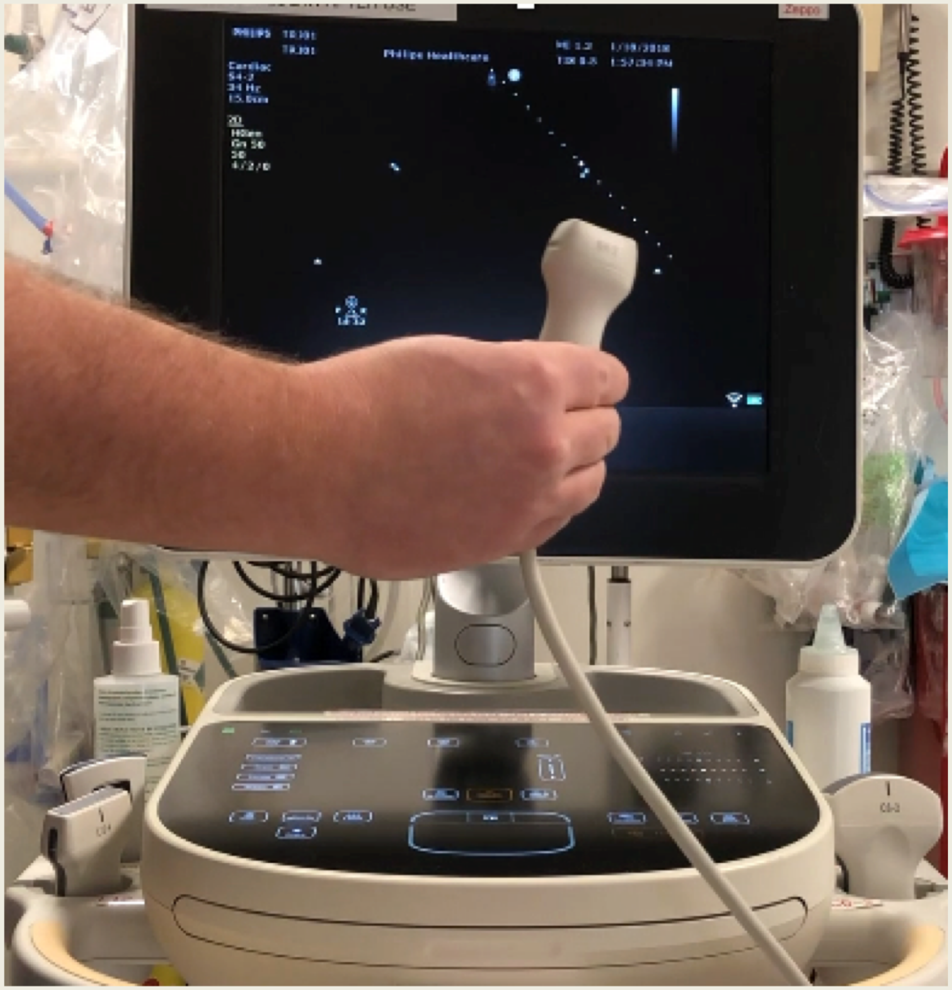
PHILIPS
Cardiac

Positioning

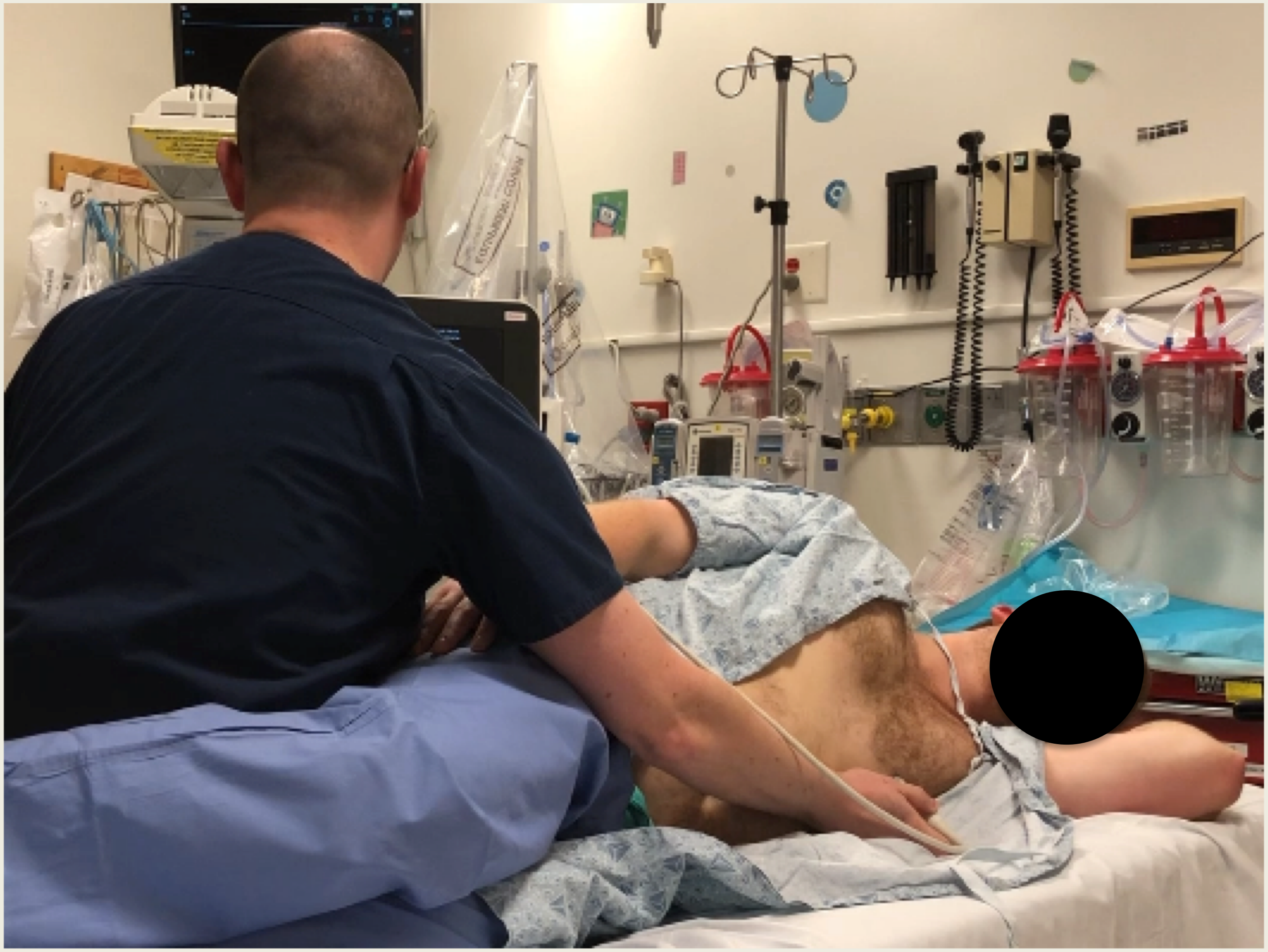

Patient Positioning

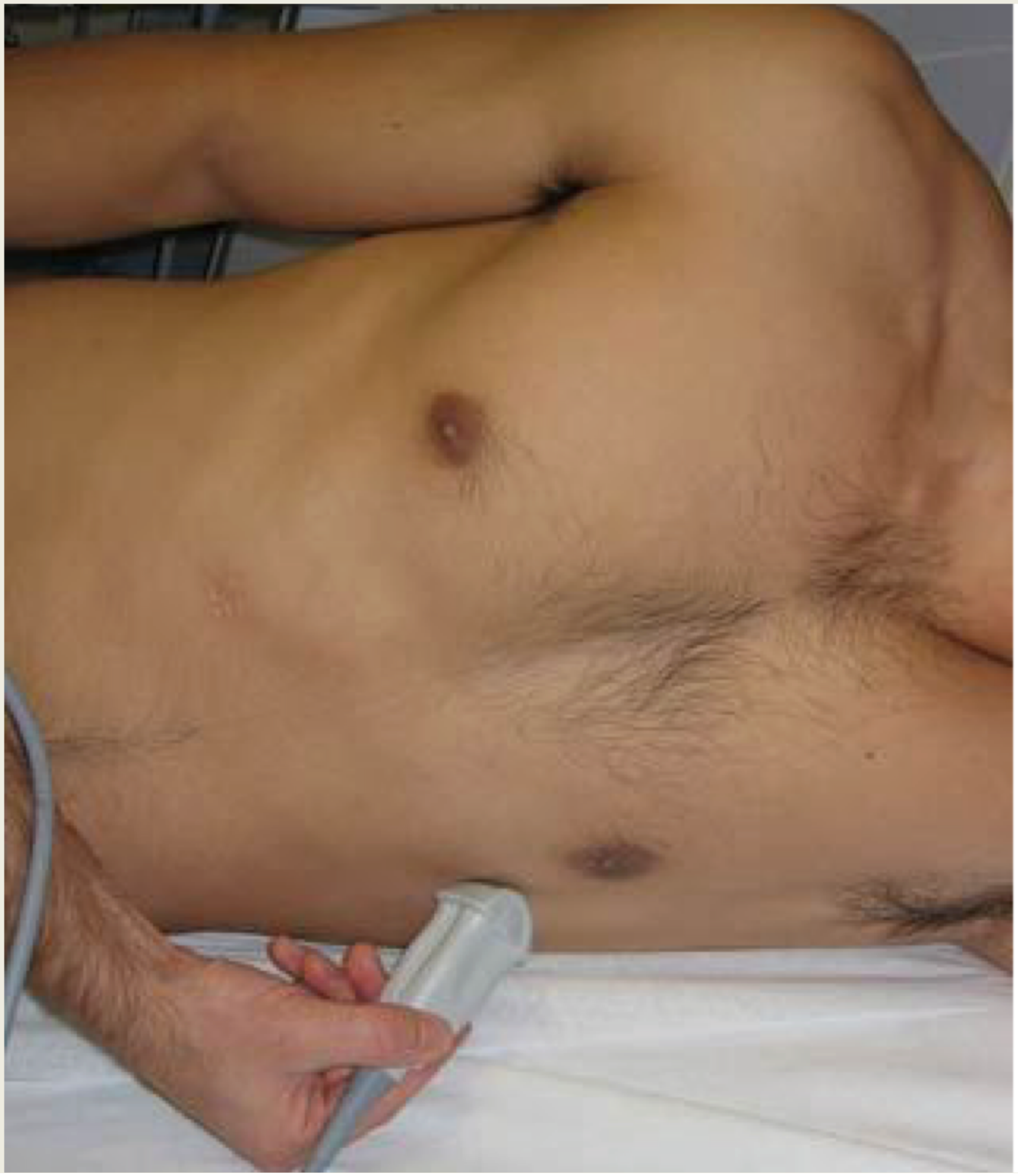

Overview

1. Apical 4 chamber
2. Color Box Positioning
3. Tricuspid Regurgitant Jet Velocity Color Signal Optimization
4. Continuous Wave Doppler
5. Measurement of TRJ

Tricuspid Regurgitant Jet Velocity Curriculum

Curricular Element	Critical Action Step
Apical 4-Chamber View	Image orientation with cardiac apex at the top of screen and left ventricle to the right of screen
	Outline of all four chambers simultaneously visualized
	Image aligned with ultrasound beam parallel to intraventricular septum and perpendicular to Tricuspid Valve
	Image saved of apical 4-chamber view
Color Box Positioning	Color box extending from the back wall of right atrium past the tricuspid valve leaflet tips
	Color box width minimized to just include tricuspid valve orifice
TRJV Color Signal Optimization	Clip saved showing a dynamic sweep through the Tricuspid Valve (anterior → posterior) or (posterior → anterior)
	Select probe position that generates maximal regurgitant color signal
Continuous Wave Doppler	Doppler cursor placed in the middle of tricuspid regurgitant color jet
	Doppler cursor aligned parallel to color jet flow
	Doppler gain adjusted to maximize waveform
	Baseline adjusted to maximize display of wave form
	Image includes three full cardiac cycles
	Image saved with continuous-wave Doppler applied

Apical 4 - Chamber

Apical 4-Chamber View

Apical 4-Chamber View	Image orientation with cardiac apex at the top of screen and left ventricle to the right of screen
	Outline of all four chambers simultaneously visualized
	Image aligned with ultrasound beam parallel to intraventricular septum and perpendicular to Tricuspid Valve
	Image saved of apical 4-chamber view

Apical 4 Chamber View

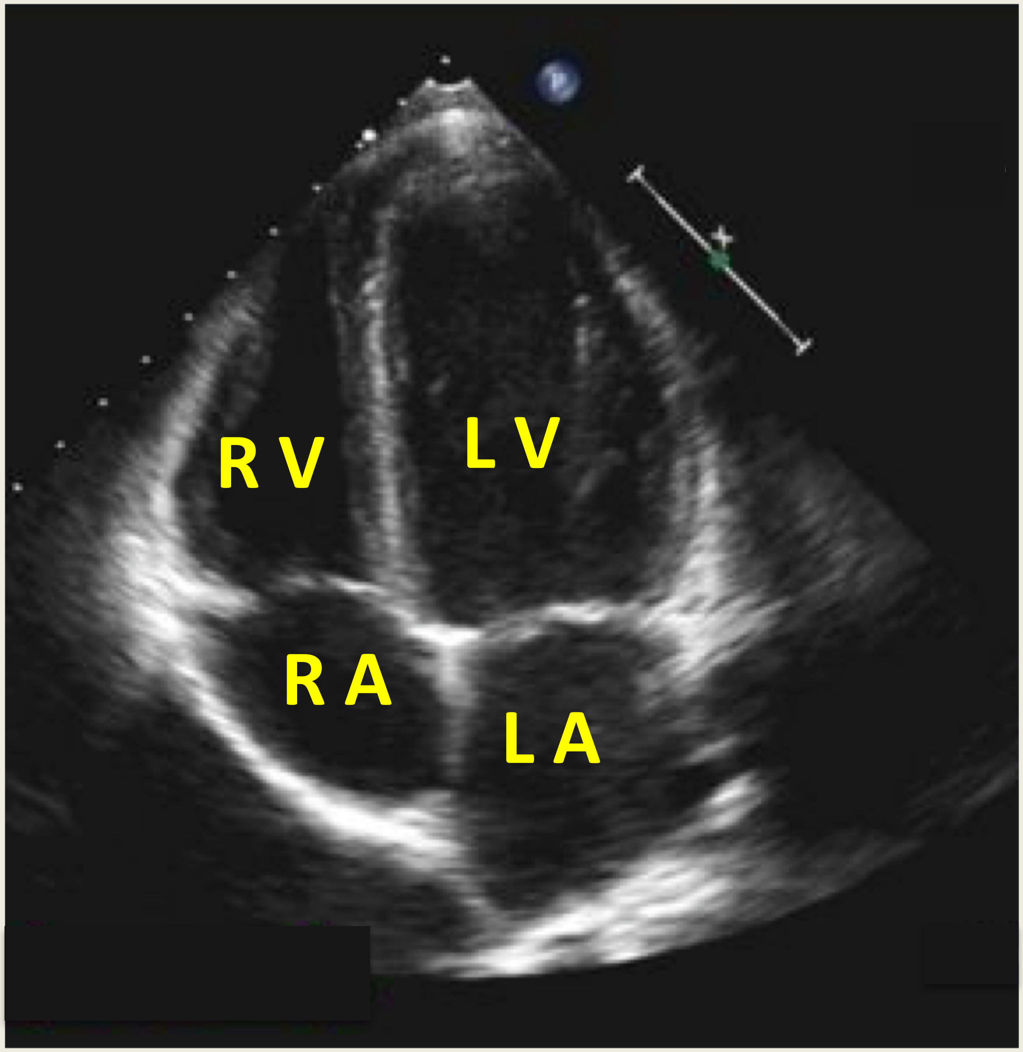

Apical 4 Chamber View

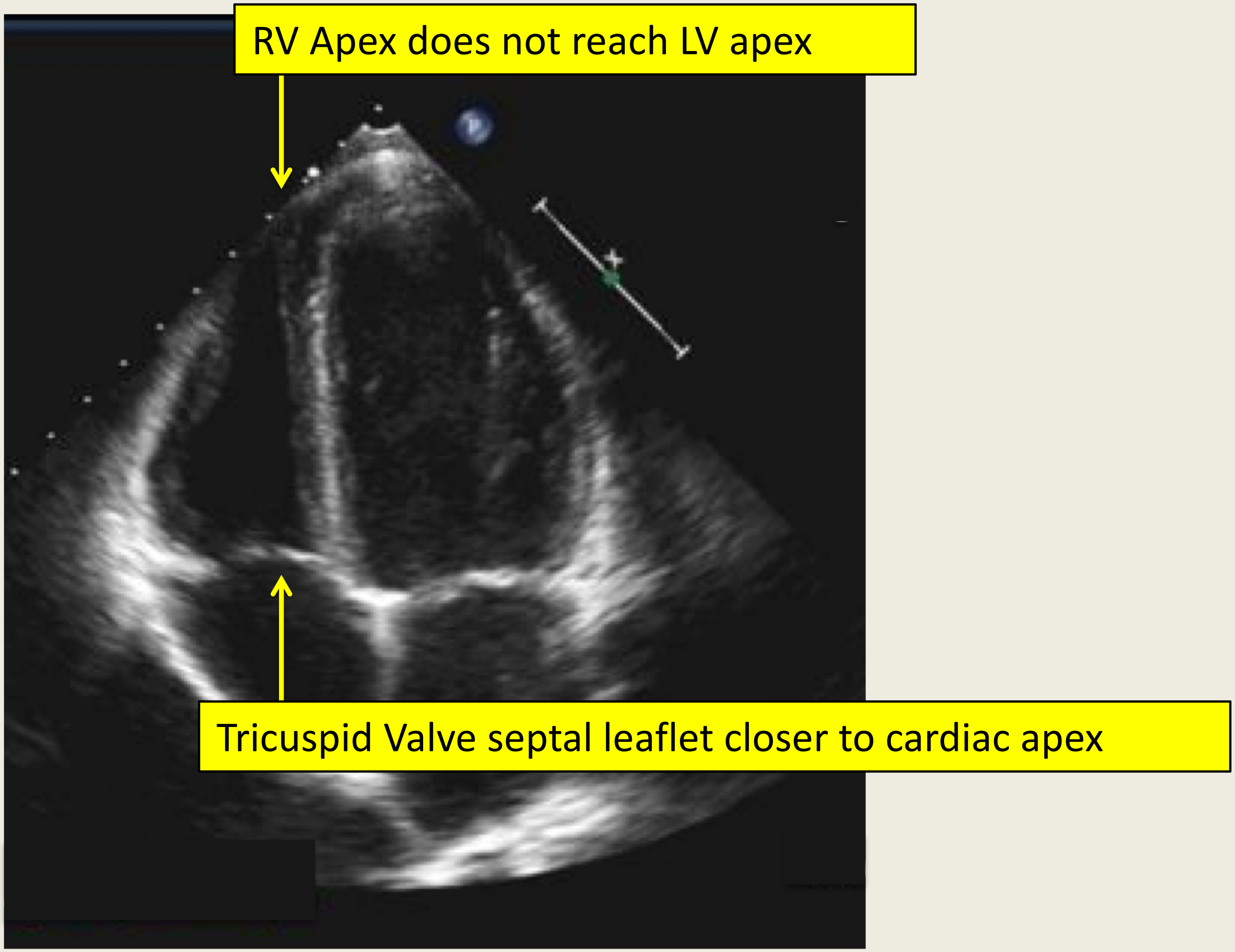

Apical 4 Chamber View

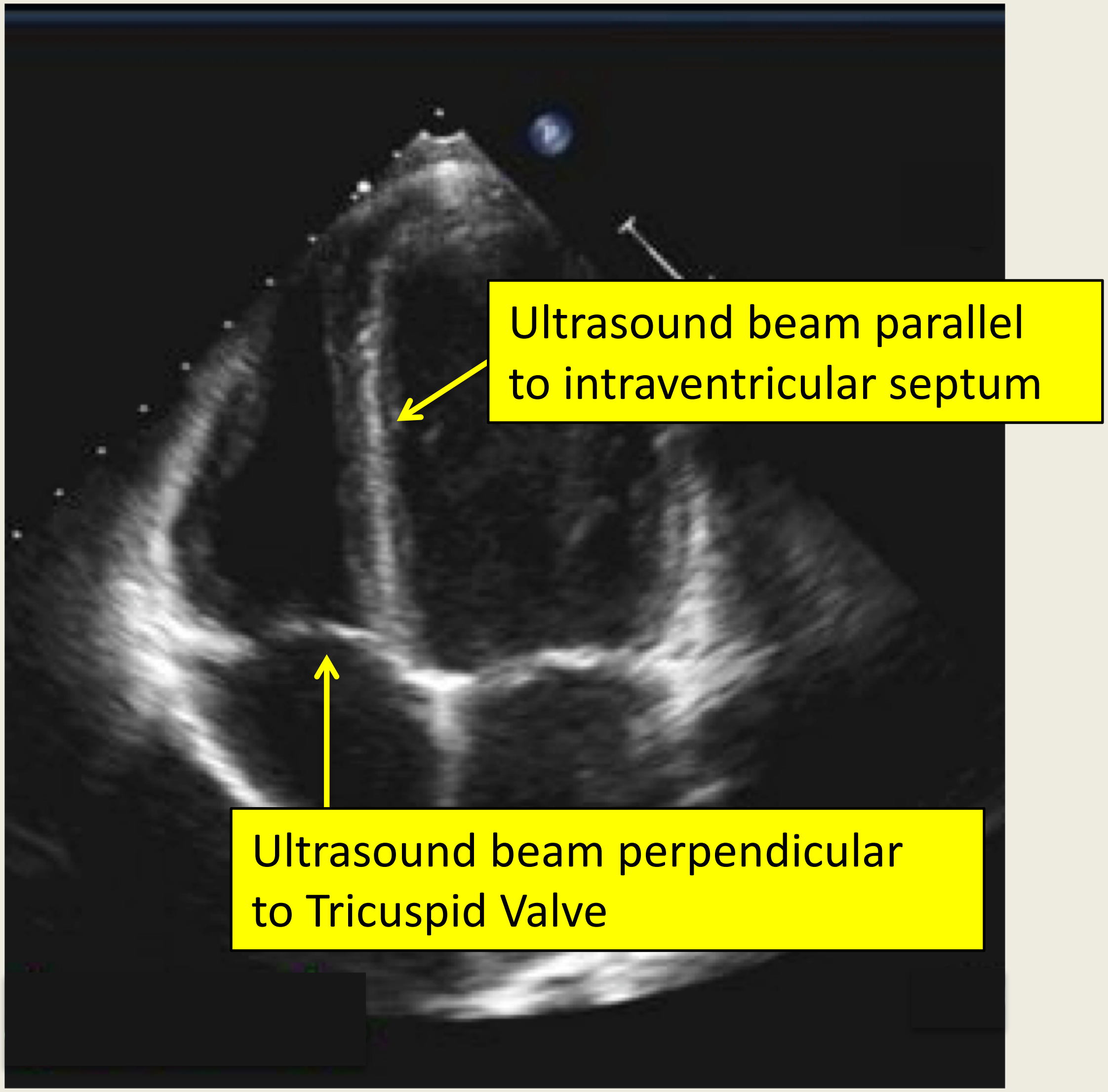

Color Box Positioning

Color Box Positioning

Color Box Positioning	Color box extending from the back wall of right atrium past the tricuspid valve leaflet tips
	Color box width minimized to just include tricuspid valve orifice

Color Applied

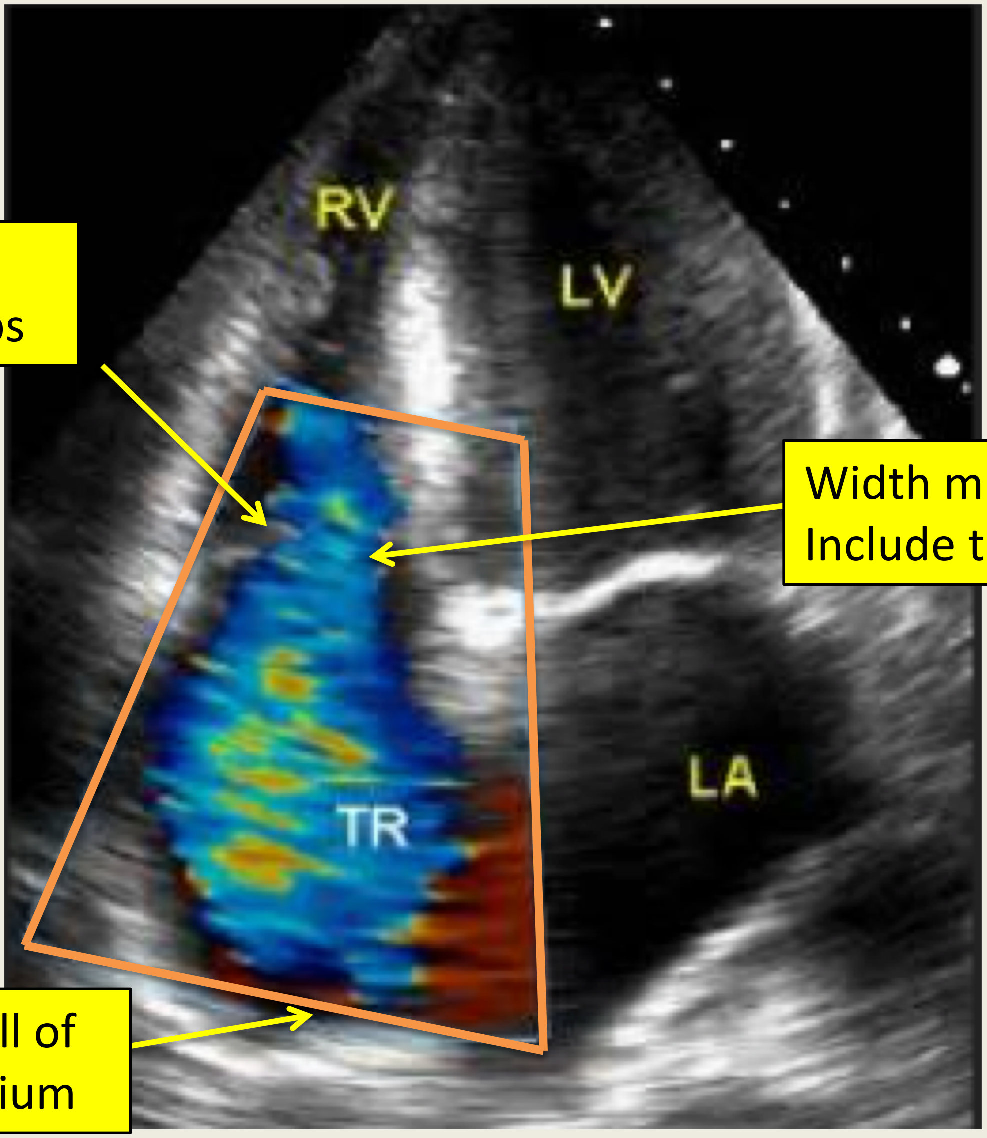

Past Tricuspid
Valve leaflet tips

Width minimized to just
Include tricuspid valve orifice

Back wall of
right atrium

Tricuspid Regurgitant Jet Velocity Color Signal Optimization

TRJV Color Signal Optimization

TRJV Color Signal Optimization	Clip saved showing a dynamic sweep through the Tricuspid Valve (anterior → posterior) or (posterior → anterior)
	Select probe position that generates maximal regurgitant color signal

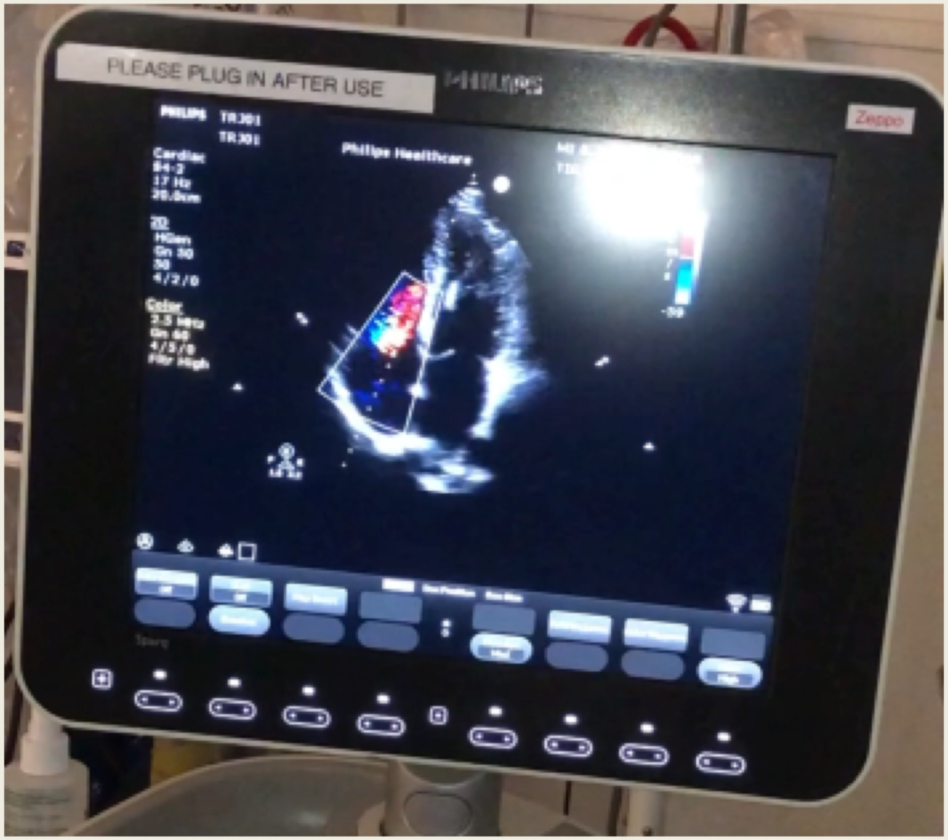
PLEASE PLUG IN AFTER USE
PHILIPS
Zeppo
Philips Healthcare
Cardiac

Continuous Wave Doppler

Continuous Wave Doppler

Continuous Wave Doppler	Doppler cursor placed in the middle of tricuspid regurgitant color jet
	Doppler cursor aligned parallel to color jet flow
	Doppler gain adjusted to maximize waveform
	Baseline adjusted to maximize display of wave form
	Image includes three full cardiac cycles
	Image saved with continuous-wave Doppler applied

Continuous Wave Doppler

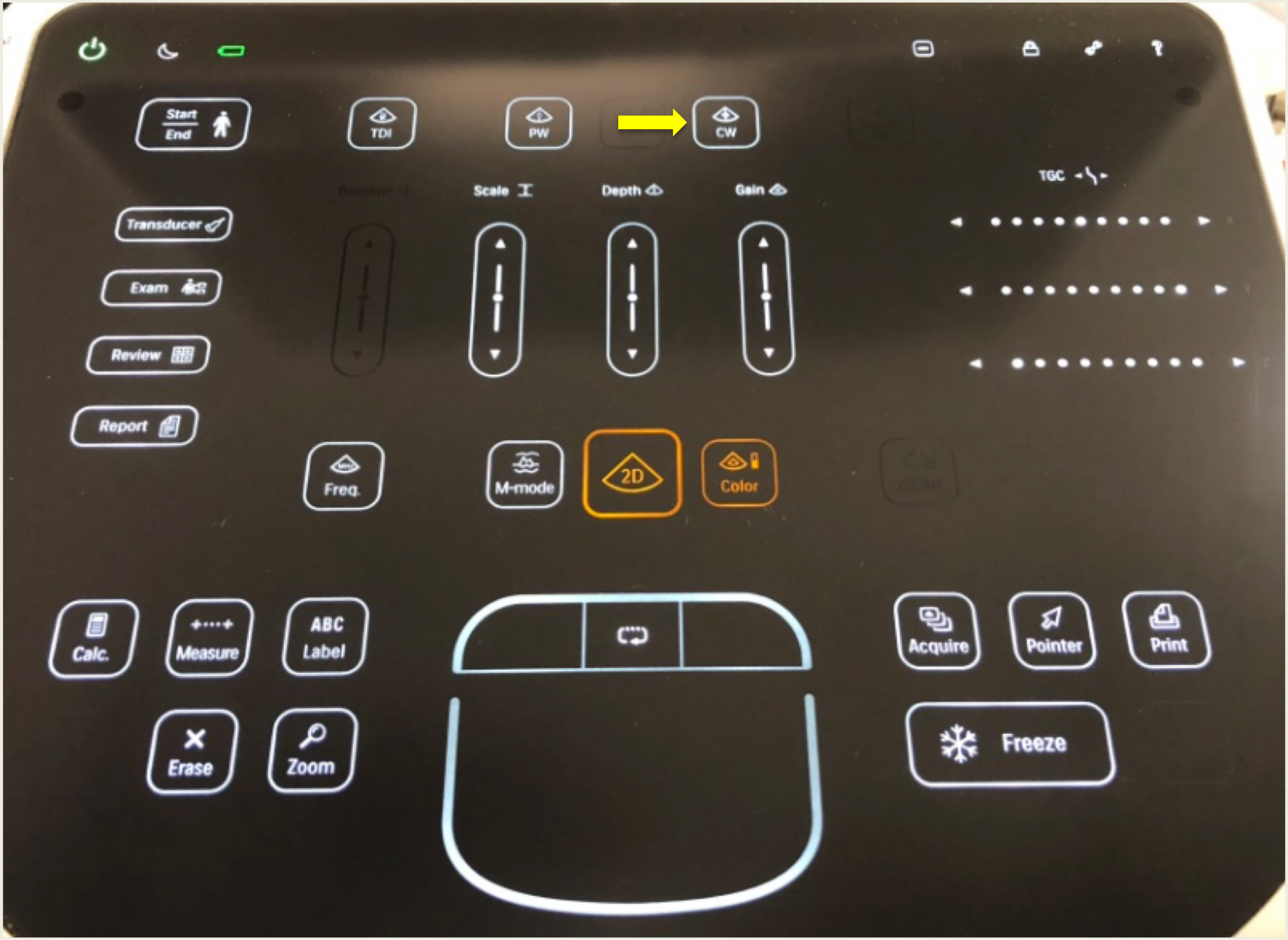

Continuous Wave Doppler

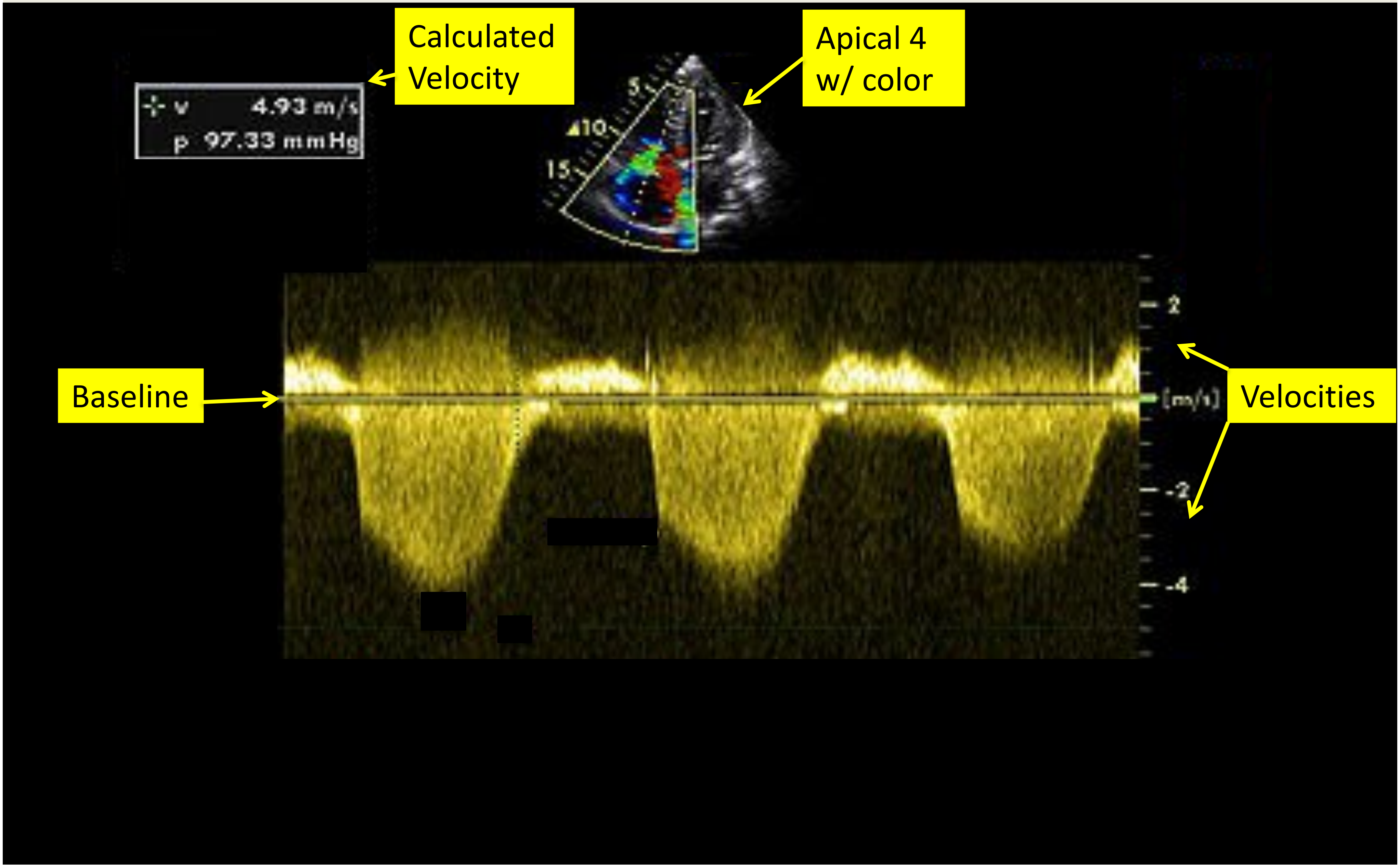

Continuous Wave Doppler

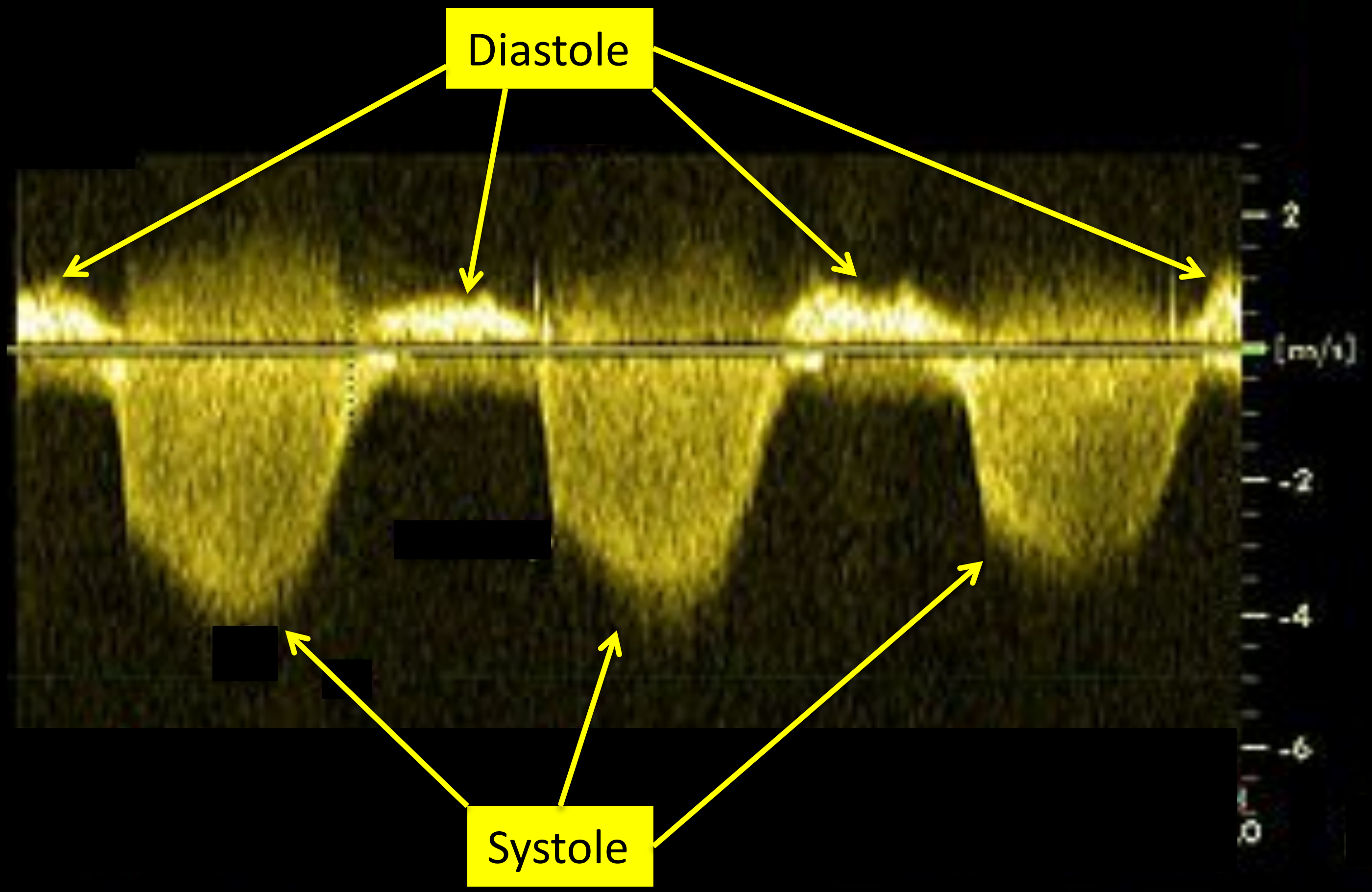

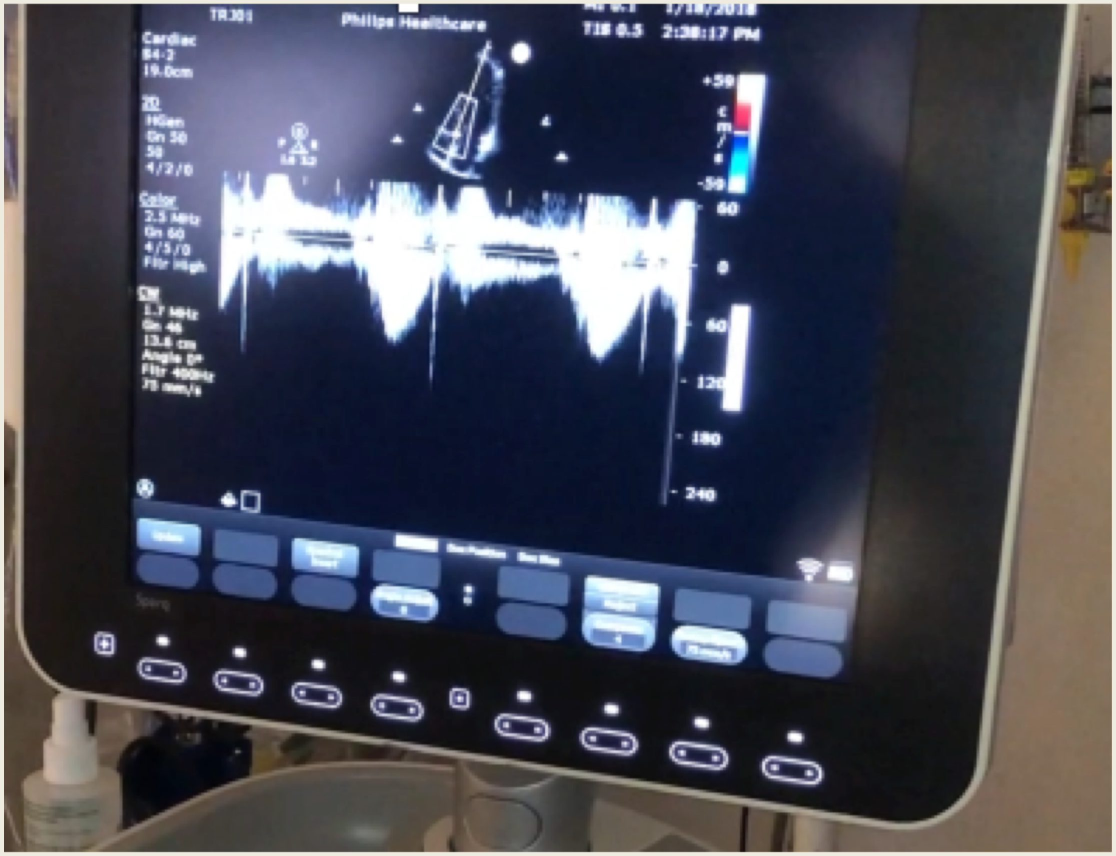
TR301
Philips Healthcare
TIS 0.5
2:38:17 PM
Cardiac
S4-2
19.0cm
2D
HGen
Gn 50
50
4/2/0
Color
2.5 MHz
Gn 60
4/5/0
Fltr High
CW
1.7 MHz
Gn 46
13.6 cm
Angle 0°
Fltr 400Hz
75 mm/s
+50
cm/s
-50
60
0
60
120
180
240

Measurement

Measurement

Measurement curser placed at the peak of Doppler waveform; feathering excluded

Clip saved with measurement caliper applied

Freeze Image / Bring Up Measurement Calipers

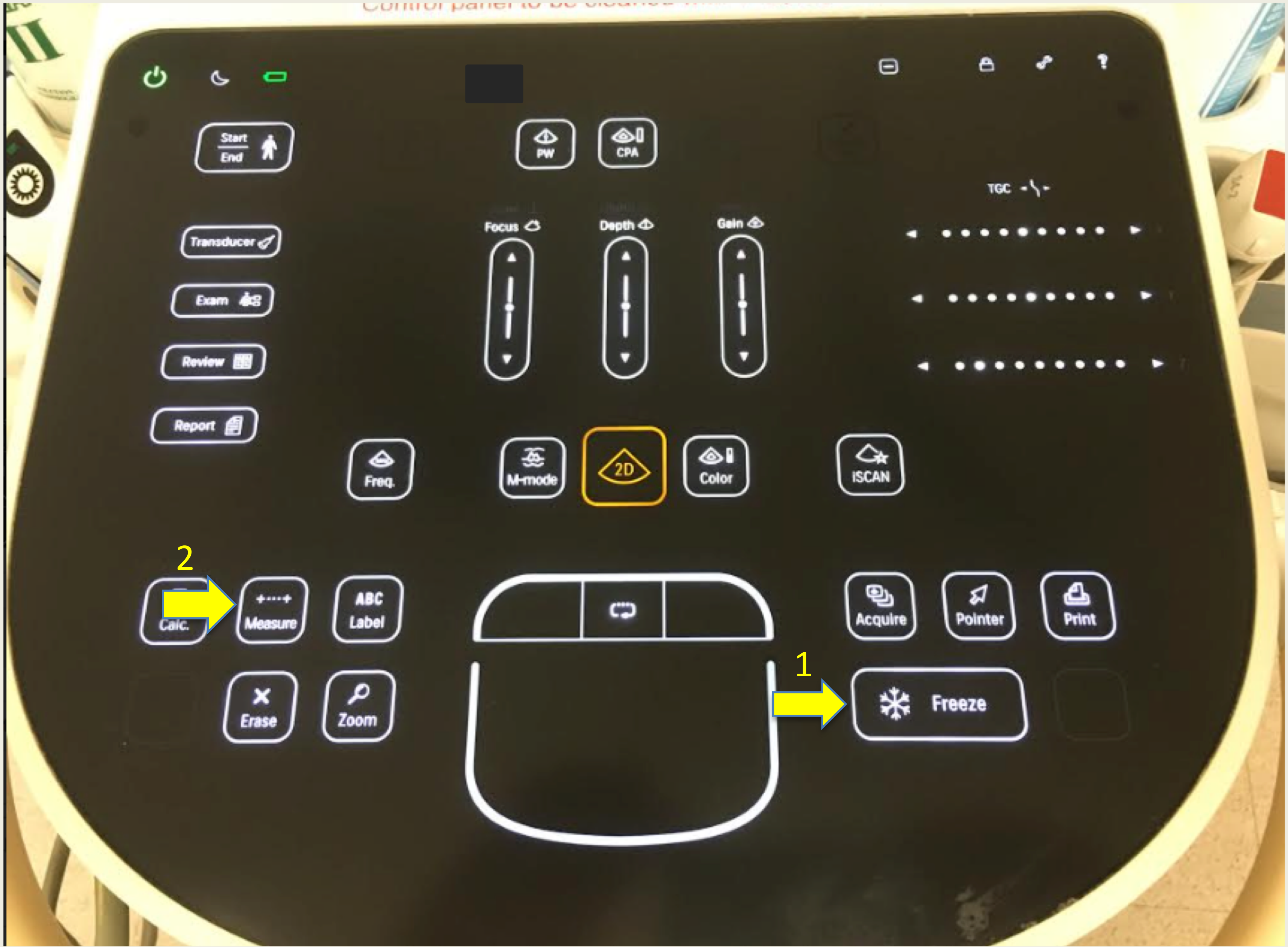

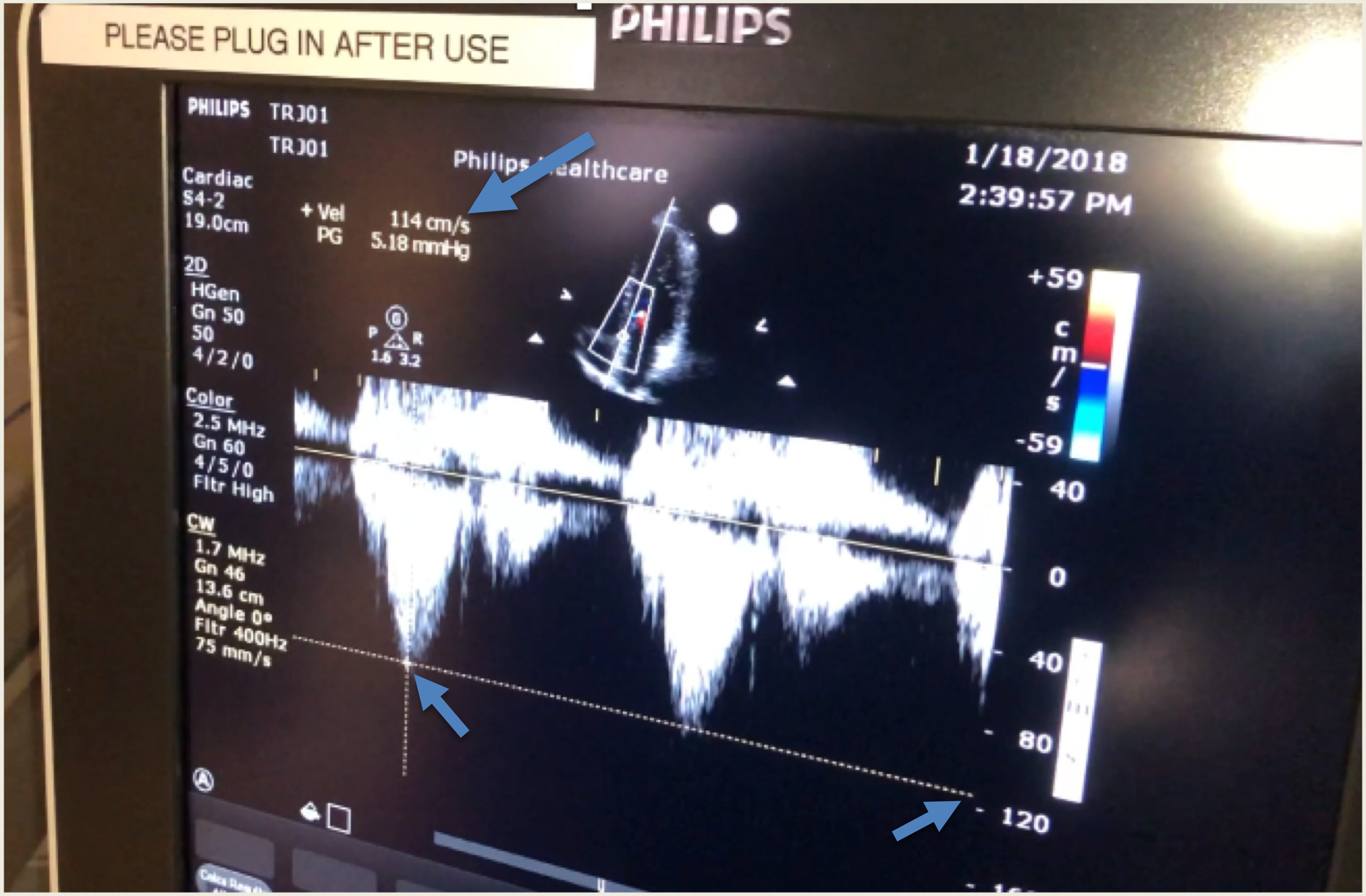
PLEASE PLUG IN AFTER USE
PHILIPS
PHILIPS TRJ01
TRJ01
Philips Healthcare
1/18/2018
2:39:57 PM
Cardiac
S4-2
19.0cm
+ Vel 114 cm/s
PG 5.18 mmHg
2D
HGen
Gn 50
50
4/2/0
Color
2.5 MHz
Gn 60
4/5/0
Fltr High
CW
1.7 MHz
Gn 46
13.6 cm
Angle 0°
Fltr 400Hz
75 mm/s
+59
cm/s
-59
40
0
40
80
120

Exclude Feathering

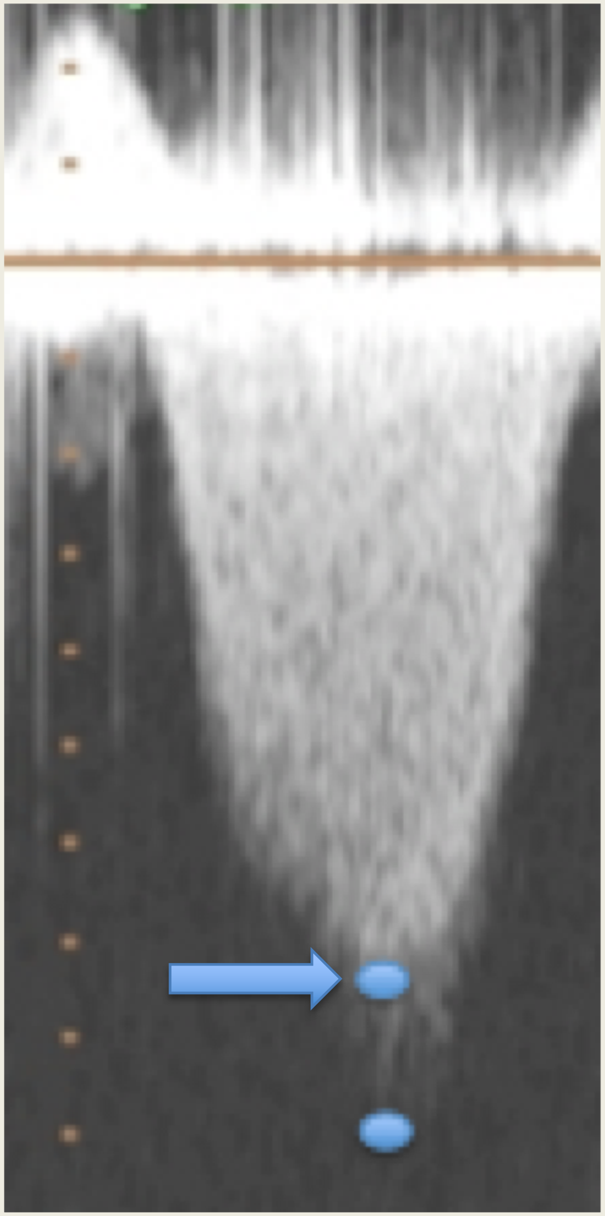

Review

1. Apical 4 chamber → SAVE Image
2. Color Applied → SAVE Clip
3. Color Signal Optimized → SAVE Clip
4. Continuous Doppler → SAVE Image
5. Measurement → SAVE Image
