# Supplementary material for: Tricuspid Regurgitant Jet Velocity Point-of-Care Ultrasound Curriculum Development and Validation
Source: POCUS J. 2021 Nov 23;6(2):88–92. doi: 10.24908/pocus.v6i2.15190 (PMC9316333; doi:10.24908/pocus.v6i2.15190)
Supplement: Supplementary Document S5 [file pocusj-06-15190-s006.pdf]

TR Jet Grading Rubric

Each point graded as Complete (1), Incomplete (0), or Not Performed (0)

1) Apical 4-chamber view (with attention to Tricuspid Valve)
   -Ultrasound beam parallel to ventricular septum and perpendicular to TV (+/- 20 degrees)
   -Entire width of TV orifice visualized with-in image

2) Color Box Dimensions
   - Color box "height" must include back wall of RA
   - Color box "height" must include TV leaflet tips
   - Color box "width" minimized to just include TV orifice

3) Color Doppler Optimization
   - Image clip showing dynamic sweep through the tricuspid valve
   - Probe position selected that maximizes color signal (probe positioning must be graded as good or excellent on poor/fair/good/excellent scale in order to qualify)

4) Doppler Acquisition
   - Doppler curser properly placed (middle of TR color jet and aligned as parallel as possible with color jet)
   - Baseline adjusted to maximize waveform display
